# A cross‐sectional study on HPV testing with type 16/18 genotyping for cervical cancer screening in 11,064 Chinese women

**DOI:** 10.1002/cam4.1060

**Published:** 2017-04-04

**Authors:** Qiongyan Wu, Xiumin Zhao, Yunfeng Fu, Xinyu Wang, Xiaofei Zhang, Xun Tian, Bei Cheng, Bingjian Lu, Xiao Yu, Suqiu Lan, Weiguo Lu, Ding Ma, Xiaodong Cheng, Xing Xie

**Affiliations:** ^1^ Department of Gynecologic OncologyWomen's HospitalSchool of MedicineZhejiang UniversityHangzhouChina; ^2^ Key Laboratory of Women's Reproductive Health of Zhejiang ProvinceHangzhouChina; ^3^ Department of PathologyWomen's HospitalSchool of MedicineZhejiang UniversityHangzhouChina; ^4^ Cancer Biology Research Center (Key laboratory of the ministry of education)Tongji HospitalTongji Medical CollegeHuazhong University of Science and TechnologyWuhanHubeiChina; ^5^ Department of GynecologyWomen & Children Healthcare Hospital of Quzhou CityQuzhouZhejiangChina; ^6^ Longyou County Maternal and Child Health‐Care CenterQuzhouZhejiangChina

**Keywords:** Cervical cancer screening, cervical cytology, cervical intraepithelial neoplasia, genotype, human papillomavirus

## Abstract

Cytology‐based cervical cancer screening is restricted because of a lack of cytologists. Thus, HPV‐based instead of cytology‐based screening may be a more suitable strategy in China. Here, we assessed the effectiveness of HPV testing (Cobas^®^ 4800 Test, Roche) and HPV‐based programs to detect high‐grade cervical intraepithelial neoplasia (CIN) or cancer compared with cytology (Thinprep, Hologic) and cytology‐based programs through a cross‐sectional study in 11,064 Chinese women aged 21–65 years who were enrolled from Longyou County in Zhejiang Province, China. The rates of HPV positivity and cytology abnormality were 9.8% and 6.1%, respectively. The HPV positivity rate had two age peaks, 21–24 (15.4%) and 60–65 (14.4%) years. According to adjusted data, HPV testing demonstrated significantly higher sensitivity and negative predictive value (NPV) than cytology for detecting CIN2 or worse (90.0% vs. 66.7%, 99.9% vs. 99.5%), and there was an acceptable specificity (91.3%) and positive predictive value (PPV, 12.5%). Furthermore, primary HPV testing with type 16/18 genotyping showed the highest sensitivity (78.6%) and NPV (99.7%) among four screening strategies, and there was similar specificity (96.8%) and PPV (23.9%) compared with co‐testing screening to detect CIN2+, while there were fewer colposcopies (4.2) and tests (106.3) performed than with co‐testing and primary cytology screening to detect a case of high‐grade CIN. The differences in effectiveness were approximately similar when CIN3+ was the identifying target. Our findings suggest that primary HPV testing with type 16/18 genotyping has a higher sensitivity and NPV, possesses optimal cost/effectiveness in the first round of screening and is a feasible strategy of cervical cancer screening for Chinese women.

## Introduction

Cytology‐based cervical cancer screening has greatly reduced the incidence and death of cervical cancer in developed countries. However, its suboptimal sensitivity and negative predictive value have not been overcome, despite the application of liquid‐based cytology techniques and the Bethesda diagnostic classification. The fact that high‐risk human papillomavirus (HPV) infection is a necessary event for cervical cancer development determines the value of HPV testing in cervical cancer screening. Compared with cytology, HPV DNA testing has a higher sensitivity and negative predictive value (NPV), providing more reassurance for women with a negative result. The American Society of Colposcopy and Cervical Pathology (ASCCP) initially recommended HPV testing as a triage for cytology diagnosed as atypical squamous cells of unknown significance (ASC‐US) in 2001 1 and further recommended HPV testing for co‐testing with cytology in 2006 2. The European Research Organization on Genital Infection and Neoplasia (EUROGIN) then proposed HPV testing as a primary screening approach in 2008 3. Since then, HPV‐based screening has been performed in many European and Asian countries. Recently, the United States revealed the baseline data and three‐year follow‐up data of ATHENA (Addressing the Need for Advanced HPV Diagnostics) in 2011–2015, which was the largest prospective clinical study of HPV‐based primary screening in the US, confirming that primary HPV testing screening was safer and more effective than primary cytology 4, 5, 6, 7. Based on those results, the US Food and Drug Administration (FDA) approved Roche Cobas HPV testing alone for cervical cancer screening in women over 25 years of age in 2014. Additionally, the ASCCP published interim guidelines in 2015 and recommended that primary HPV testing screening be considered as an alternative to current cytology‐based cervical cancer screening methods 8.

As the largest developing country, China possesses the most cervical cancer patients with 98,900 new cases and 30,500 death cases in 2015 9. Because of a severe lack of cytologists, cytology‐based cervical cancer screening methods are restricted in China as well as in other developing countries. Compared with other HPV tests, Cobas^®^ HPV testing using the real‐time fluorescent PCR technique can simultaneously detect HPV 16/18 and another 12 high‐risk HPV genotypes. With HPV 16/18 genotyping, the strategy of primary HPV screening with type 16/18 genotyping triage further reduces the necessary cytology tests and dependence on cytologists, which facilitates cervical cancer screening in developing countries. However, there are still no up‐to‐date data on the suitability of HPV testing with type 16/18 genotyping in Chinese women. Therefore, we assessed the effectiveness of HPV testing with type 16/18 genotyping and HPV‐based program to detect high‐grade cervical intraepithelial neoplasia (CIN) or cancer in 11,064 Chinese women through a population‐based cross‐sectional study. The aim of the study is to provide evidence of the suitability of an HPV‐based screening strategy in China.

## Materials and Methods

### Study population

This study is a prospective population‐based cancer screening trial. We chose Longyou County in Zhejiang Province, China as a candidate screening locale. A total of 332 administrative villages and communities were randomly selected, and large‐scale cervical cancer screening was not performed in the past three years in these communities. All women were recruited by local community staff and doctors in the Longyou County Maternal and Child Health‐Care Center according to household registry. Approximately 20% of women did not respond to the screening invitation because of outgoing for work or study, and other reasons. A total of 11,356 women aged 21–65 years participated in this cervical cancer screening program. All participants had a sexual history.

Those women were excluded: pregnancy or within 6 weeks or less of the post‐partum period; previous total hysterectomy; a history of CIN or worse, vulvar intraepithelial neoplasia or worse, or vaginal intraepithelial neoplasia or worse; a history of other malignancies; a history of cervical cancer screening or physical cervical therapy in the past three years; serious autoimmune disease or uremia; and vaccinated or planned to vaccinate against HPV infection in the near future.

The study was in line with the 2013 Declaration of Helsinki and was approved by the Ethics Committee of Women's Hospital, Zhejiang University School of Medicine.

### Study protocol

Each woman who met the inclusion criteria without meeting the exclusion criteria signed the informed consent form, provided a brief medical history, and underwent speculum examination by a gynecologist. According to instruction, samples of cervical exfoliate cells were collected using a cytology brush (Hologic, Bedford, MA) and stored in the tubes with preservation solution for the Thinprep cytology test (TCT, Hologic, Bedford, MA) and HPV DNA test (Cobas^®^ 4800 Test, Roche Molecular Systems, Pleasanton, CA), respectively. All samples were collected during April to May of 2015.

The results of HPV testing were divided into the following: HPV‐, HPV16/18+ (result positive for either genotype 16 or 18, with or without 12 other types), and HPV non‐16/18+ (result negative for genotype 16/18 and positive for 1 or more of 12 other high‐risk types). Cytology slides were read by two pathologists of our hospital, and 5674 cases were read with computer‐aided reading (Hologic, MA) prior to examination by cytologists. Cytology results were reported according to the Bethesda 2014 classification 10. The cytology diagnoses were divided into negative for intraepithelial lesion or malignancy (NILM), atypical cells of undetermined significance (ASC‐US), low‐grade squamous intraepithelial lesion (LSIL), atypical squamous cells–cannot exclude high‐grade squamous intraepithelial lesion (ASC‐H), high‐grade squamous intraepithelial lesion (HSIL), atypical glandular cells (AGC), and cervical cancer cells. If the diagnoses given by two cytologists were concordant, they were reported as the cytology diagnosis; otherwise, a third pathologist was consulted to reach a consensus diagnosis (2 of 3 agreements).

**Figure 1:**
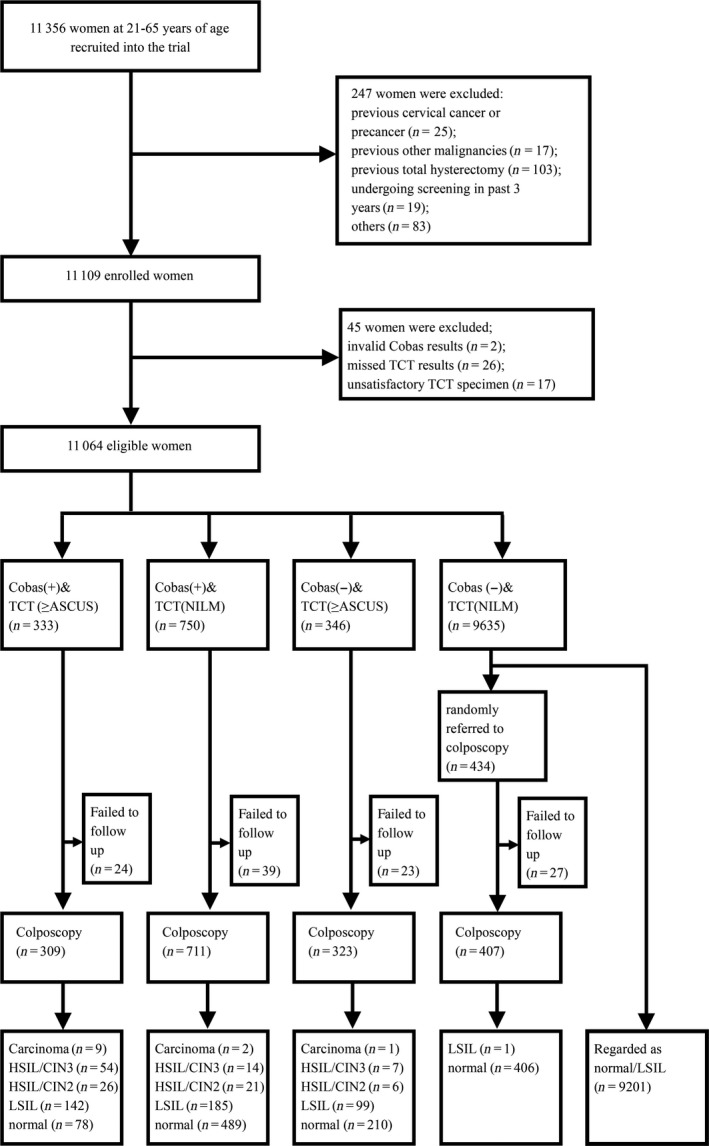
Flowchart of the screening profile.

All women with positive HPV testing or abnormal cytology (ASC‐US or worse) were referred to colposcopy; meanwhile, 4.5% of women with both negative tests were randomly selected and referred to colposcopy. Colposcopy was performed by colposcope specialists of our hospital. A woman underwent further cervical biopsy and/or endocervical curettage (ECC) if her colposcopy diagnosis was a high‐grade lesion or a non‐high‐grade lesion with one of the following conditions: cytology LSIL or worse; cytology ASC‐US and HPV+; HPV 16/18+; AGC; and unsatisfactory colposcopy. Tissues were paraffin embedded and slides were routinely HE stained. Two pathologists at our hospital separately made the diagnosis. If the diagnoses were concordant, they were reported as the pathologic diagnosis; otherwise, a panel of pathologists was consulted to reach a consensus diagnosis. The pathologic diagnosis standard was the 2014 WHO Classification of Tumors of the Female Genital Tract 11, 12. The histological diagnoses of cervical lesions were divided into normal, low‐grade squamous intraepithelial lesion (LSIL/CIN1, including the condylomatous variant), high‐grade squamous intraepithelial lesion (HSIL)/CIN2, HSIL/CIN3 (including adenocarcinoma *in situ*), and carcinoma (squamous cell carcinoma or adenocarcinoma). Due to ethical considerations, most of the women with both negative HPV and cytology results were not referred to colposcopy and biopsy/ECC but were all regarded as LSIL or less. All women with pathologic abnormalities were processed according to the newest ASCCP guidelines 13. The screening process is shown in Figure 1. HPV testing, cytology, and pathologic examination were performed with blinding to the results of each test.

**Figure 2:**
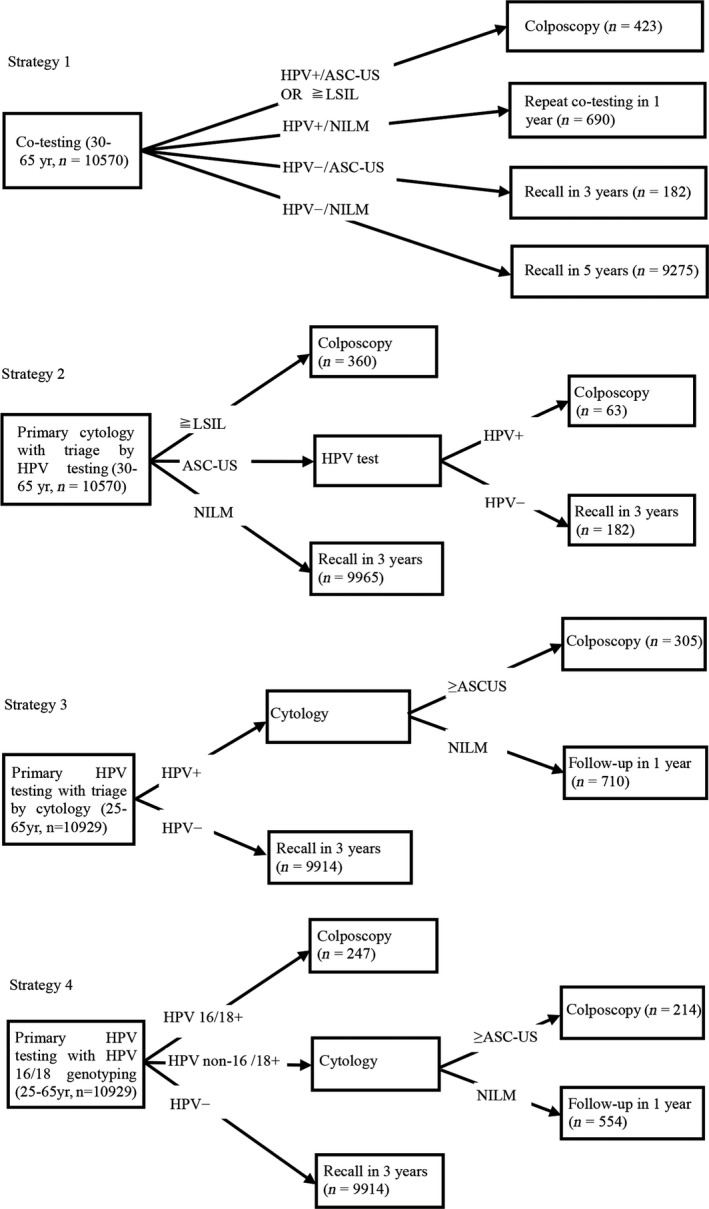
Algorithms of four strategies to detect high‐grade CIN or worse.

The study initially assessed the effectiveness of HPV testing, with cytology testing as a control, for identifying high‐grade CIN and then compared the effectiveness of four strategies that are currently used for cervical cancer screening. Strategy 1 (Co‐testing) primarily screens women with both cytology and HPV testing, and then refers those with cytology ASC‐US/HPV+ or LSIL or worse to colposcopy. Strategy 2 (Primary cytology with triage by HPV testing) primarily screens women with cytology alone and then refers those with cytology LSIL or worse to colposcopy, triages those with ASC‐US by reflex HPV testing, and refers HPV+ women to colposcopy. Strategy 3 (Primary HPV testing with triage by cytology) primarily screens women with HPV testing alone, triages HPV+ women with cytology, and refers those with cytology ASC‐US or worse to colposcopy. Strategy 4 (Primary HPV testing with type 16/18 genotyping) primarily screens women with HPV testing plus type 16/18 genotyping, refers HPV 16/18+ women to colposcopy, triages HPV non‐16/18+ women with cytology and refers those with cytology ASC‐US or worse to colposcopy. A detailed flow chart is shown in Figure 2.

### Statistical analysis

Abnormal rates of cytology and HPV testing were calculated based on all participants with cytology and HPV testing results. The effectiveness of detecting high‐grade CIN was assessed using the sensitivity, specificity, positive predictive value (PPV), NPV, positive likelihood ratio (PLR), and negative likelihood ratio (NLR). Both raw and adjusted data were analyzed. When adjusted data were analyzed, the numbers of participants with pathological examination diagnosed as CIN2 or worse were used as a positive base to calculate the likely number of high‐grade CIN cases that would have been found if all participants were referred to colposcopy for different cytology and HPV testing results. For screening strategies, the sensitivity, specificity, PPV, and NPV as well as the numbers of colposcopy cases and of screening tests needed to detect one case of high‐grade CIN were calculated. All data were analyzed by SPSS 19.0 and medcalc 15.6 software. A *P* value less than 0.05 (two‐sided) was considered statistically significant.

## Results

### Comparison between HPV testing and cytology to identify high‐grade CIN

Of 11,356 participants, 247 women met the exclusion criteria and 45 had invalid HPV results/invalid or unsatisfactory cytology, and they were excluded. As a result, 11,064 women were included in the analysis.

**Figure 3:**
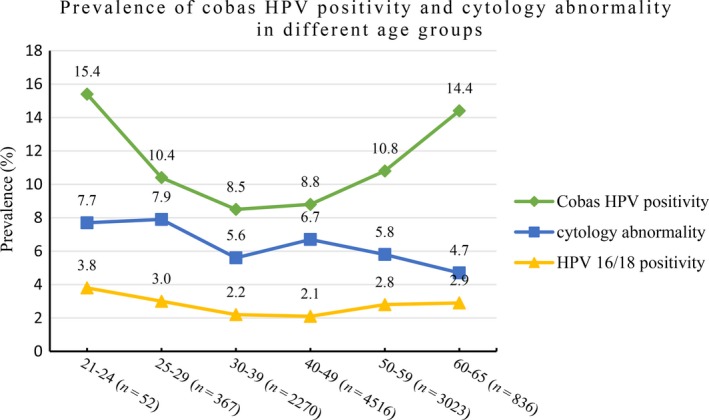
Prevalence of HPV positivity and cytological abnormality in different age groups. HPV positivity means positive for any of 14 high‐risk HPV types. Cytological abnormality means atypical squamous cells of undetermined significance (ASC‐US) or worse. HPV16/18 positivity means positive for either genotype 16 or 18, with or without 12 other types.

The total positivity rate of HPV testing was 9.8%. The positivity rates of HPV 16, HPV 18 and other high‐grade genotypes were 1.8%, 0.6% and 7.4%, respectively. The HPV positivity rate had two age peaks, 21–24 (15.4%) and 60–65 (14.4%) years, as shown in Figure 3. The total abnormal cytology rate was 6.1%, with frequencies of ASC‐US of 2.5%, AGC of 0.05%, LSIL of 2.4%, ASC‐H of 0.6%, HSIL of 0.5%, and cervical cancer of 0.1%, respectively. A total of 1750 women were referred to colposcopy; of those, 567 were pathologically abnormal, including 427 cases with CIN1, 53 with CIN2, 75 with CIN3 (including 2 adenocarcinomas in situ), and 12 with cervical squamous carcinoma.

The effectiveness of cytology and HPV testing to identify high‐grade CIN is shown in Table 1. Before verification bias correction, compared with cytology (LSIL or worse), HPV testing had a higher sensitivity (90.0% vs. 66.4%, *P *=* *0.000) and NPV (98.1% vs. 96.6%, *P *=* *0.050), while it had a significantly lower specificity (44.5% vs. 82.4%, *P *=* *0.000), for detecting CIN2+. After verification of bias correction, the sensitivities of HPV testing and cytology were 90.0% and 66.7%, but the specificities were 91.3% and 97.2%, respectively, and the difference was significant. The AUC of HPV testing was larger than that of cytology (0.91 vs. 0.82) for detecting CIN2+. With CIN3+ as the identifying target, HPV testing and cytology demonstrated similar results.

**Table 1:** Performance of HPV and cytology for identifying women with CIN2+ or CIN3+

	Crude data		*P* value	Adjusted data		*P* value
	HPV	Cytology (≥LSIL)		HPV	Cytology (≥LSIL)	
CIN2+						
Sensitivity	126/140 (90.0%, 83.8–94.4)	93/140 (66.4%, 58.0–74.2)	*P *=* *0.000	135/150 (90.0%, 84.0–94.3)	100/150 (66.7%, 58.5–74.1)	*P *=* *0.000
Specificity	716/1610 (44.5%, 42.0–46.9)	1326/1610 (82.4%, 80.4–84.2)	*P *=* *0.000	9966/10914 (91.3%, 90.8%–91.8%)	10611/10914 (97.2%, 96.9%–97.5%)	*P *=* *0.000
YI	0.35	0.49		0.81	0.64	
PPV	126/1020 (12.4%, 10.4–14.5)	93/377 (24.7%, 20.4–29.3)	*P *=* *0.000	135/1083 (12.5%, 10.6–14.6)	100/403 (24.8%, 20.7–29.3)	*P *=* *0.000
NPV	716/730 (98.1%, 96.8–99.0)	1326/1373 (96.6%, 95.5–97.5)	*P *=* *0.050	9966/9981 (99.9%, 99.8–99.9)	10611/10661 (99.5%, 99.4–99.7)	*P *=* *0.000
PLR	1.62 (1.51–1.74)	3.77 (3.22–4.41)		10.36 (9.56–11.23)	24.0 (20.5–28.1)	
NLR	0.22 (0.14–0.37)	0.41 (0.32–0.52)		0.11 (0.07–0.18)	0.34 (0.27–0.43)	
CIN3+						
Sensitivity	79/87 (90.8%, 82.7–96.0)	64/87 (73.6%, 63.0–82.5)	*P *=* *0.009	84/92 (91.3%, 83.6–96.2)	68/92 (73.9%, 63.7–82.5)	*P *=* *0.006
Specificity	722/1663 (43.4%, 41.0–45.8)	1350/1663 (81.2%, 79.2–83.0)	*P *=* *0.000	9973/10972 (90.9%, 90.3–91.4)	10637/10972 (97.0%, 96.6–97.3)	*P *=* *0.000
YI	0.34	0.55		0.82	0.71	
PPV	79/1020 (7.8%, 6.2–9.6)	64/377 (17.0%, 13.3–21.2)	*P *=* *0.000	84/1083 (7.8%, 6.2–9.5)	68/403 (16.9%, 13.4–20.9)	*P *=* *0.000
NPV	722/730 (98.9%, 97.9–99.5)	1350/1373 (98.3%, 97.5–98.9)	*P *=* *0.294	9973/9981 (99.9%, 99.8–99.9)	10637/10661 (99.8%, 99.7–99.9)	*P *=* *0.008
PLR	1.60 (1.48–1.74)	3.91 (3.33–4.59)		10.03 (9.20–10.93)	24.21 (20.61–28.43)	
NLR	0.21 (0.11–0.41)	0.33 (0.23–0.46)		0.10 (0.05–0.19)	0.27 (0.19–0.38)	

Data were presented as n/N (%, 95%CI). CIN2 +  means cervical intraepithelial neoplasia grade 2 or worse. CIN3 +  means cervical intraepithelial neoplasia grade 3 or worse. YI means Youden's index. PPV means positive predictive value. NPV means negative predictive value. PLR means positive likelihood ratio. NLR means negative likelihood ratio. Data were adjusted for verification bias.

All subjects were divided into three age‐groups: 21–24 years, 25–49 years, and 50–65 years. We compared the effectiveness of HPV testing for detecting high‐grade CIN in different age‐groups. The 21‐ to 24‐year group was not included in the statistics because there were too few samples. With CIN2+ as the identifying target, after verification bias correction, the sensitivity of HPV testing in the 50‐ to 65‐year group was higher than that in the 25‐ to 49‐year group (97.6% vs. 87.2%), but the effect was not significant, and the NPV was similar (99.97% vs. 99.8%). However, the specificity in the 50‐ to 65‐year group was significantly lower than that in the 25‐ to 49‐year group (89.3% vs. 92.4%), and the PPV in the 50‐ to 65‐year group was significantly lower than that in the 25‐ to 49‐year group (9.0% vs. 15.1%). The differences in effectiveness between the 25‐ to 49‐ and 50‐ to 65‐year groups were similar when CIN3+ was the identifying target, as shown in Table 2.

**Table 2:** Performance of HPV in the 25‐ to 49‐year group and 50‐ to 65‐year group to identify women with CIN2+ or CIN3+

	Crude data		*P* value	Adjusted data		*P* value
	25‐ to 49‐year group	50‐ to 65‐year group		25‐ to 49‐year group	50‐ to 65‐year group	
CIN2+						
Sensitivity	89/102 (87.3%, 79.2–93.0)	37/38 (97.4%, 86.2–99.9)	*P *=* *0.112	95/109 (87.2%, 79.4–92.8)	40/41 (97.6%, 87.1–99.9)	*P *=* *0.070
Specificity	511/1017 (50.3%, 47.1–53.4)	203/586 (34.6%, 30.8–38.7)	*P *=* *0.000	6511/7044 (92.4%, 91.8–93.0)	3411/3818 (89.3%, 88.3–90.3)	*P *=* *0.000
PPV	89/595 (15.0%, 12.2–18.1)	37/420 (8.8%,6.3–11.9)	*P *=* *0.003	95/628 (15.1%,12.4–18.2)	40/447 (9.0%,6.5‐12.0)	*P *=* *0.003
NPV	511/524 (97.5%, 95.8–98.7)	203/204 (99.5%, 97.3–99.99)	*P *=* *0.128	6511/6525 (99.8%, 99.6–99.9)	3411/3412 (99.97%, 99.84–100.0)	*P *=* *0.024
CIN3+						
Sensitivity	52/59 (88.1%, 77.1–95.1)	27/28 (96.4%, 81.7–99.9)	*P *=* *0.428	55/62 (88.7%, 78.1–95.3)	29/30 (96.7%, 82.8–99.9)	*P *=* *0.266
Specificity	517/1060 (48.8%, 45.7–51.8)	203/596 (34.1%, 30.3–38.0)	*P *=* *0.000	6518/7091 (91.9%, 91.3–92.5)	3411/3829 (89.1%, 88.1–90.1)	*P *=* *0.000
PPV	52/595 (8.7%, 6.6–11.3)	27/420 (6.4%, 4.3–9.2)	*P *=* *0.176	55/628 (8.8%, 6.7–11.3)	29/447 (6.5%, 4.4–9.2)	*P *=* *0.172
NPV	517/524 (98.7%, 97.3–99.5)	203/204 (99.5%, 97.3–99.99)	*P *=* *0.454	6518/6525 (99.9%, 99.8–99.96)	3411/3412 (99.97%, 99.84–100.0)	*P *=* *0.278

Data were presented as n/N (%, 95%CI). CIN2+ means cervical intraepithelial neoplasia grade 2 or worse. CIN3+ means cervical intraepithelial neoplasia grade 3 or worse. PPV means positive predictive value. NPV means negative predictive value. Data were adjusted for verification bias.

### Comparison among four screening strategies to identify high‐grade CIN

**Figure 4:**
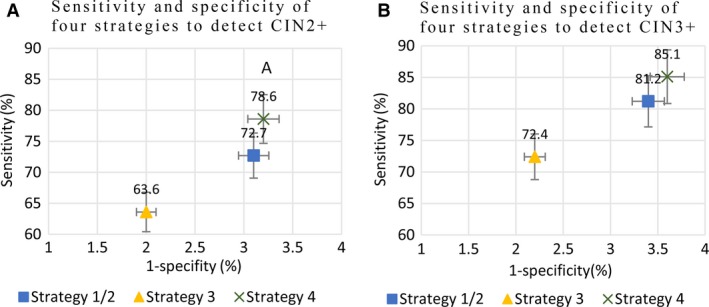
Scatter‐plot of sensitivity and specificity of the four strategies for CIN2+ (A) and CIN3+ (B). Strategy 1/2 represented Strategy 1 and 2 which had the same sensitivity and specificity. Bars represented 95% confidence intervals.

As shown in Table 3 and Figure 4, screening Strategy 1 as co‐testing demonstrated an optimal effectiveness, with a sensitivity of 72.7% and specificity of 96.9%, for identifying high‐grade CIN, but it consumed maximum tests (220.2) and colposcopies (4.4) to detect one case of high‐grade CIN. Compared with Strategy 1, Strategy 2 had the same sensitivity, specificity and number of performed colposcopies to detect one case of high‐grade CIN, but it required nearly half of the tests to detect one case of high‐grade CIN. Strategy 3 had the highest specificity (98.0%) and PPV (29.2%) with the lowest number of performed colposcopies (3.4) to detect one case of high‐grade CIN. But it had the lowest sensitivity (63.6%) and NPV (99.5%) of the four strategies and required more performed tests (134.2) than Strategy 2 to detect one case of high‐grade CIN. Strategy 4 using primary HPV testing with type 16/18 genotyping demonstrated the highest sensitivity (78.6%) and NPV (99.7%) of the four strategies, and it had a similar specificity (96.8%), PPV (23.9%) and number of performed colposcopies (4.2) as strategies 1 and 2 to detect one case of high‐grade CIN.

**Table 3:** Outcomes of four screening strategies for detecting high‐grade CIN

Strategy	Sensitivity,%	Specificity,%	PPV,%	NPV,%	Colposcopies to detect 1 case, *n*(colposcopies performed/case identified)	Tests to detect 1 case, *n*(cytology/HPV)
CIN2+						
Strategy1	72.7	96.9	22.7	99.6	4.4 (423/96)	220.2 (110.1/110.1)
Strategy2	72.7	96.9	22.7	99.6	4.4 (423/96)	112.7 (110.1/2.6)
Strategy3	63.6	98.0	29.2	99.5	3.4 (305/89)	134.2 (11.4/122.8)
Strategy4	78.6	96.8	23.9	99.7	4.2 (461/110)	106.3 (7.0/99.4)
CIN3+						
Strategy1	81.2	96.6	16.3	99.8	6.1 (423/69)	306.4 (153.2/153.2)
Strategy2	81.2	96.6	16.3	99.8	6.1 (423/69)	156.7 (153.2/3.6)
Strategy3	72.4	97.8	20.7	99.8	4.8 (305/63)	189.6 (16.1/173.5)
Strategy4	85.1	96.4	16.1	99.9	6.2 (461/74)	158.1 (10.4/147.7)

Data were presented as n/N (%, 95%CI). CIN2 +  means cervical intraepithelial neoplasia grade 2 or worse. CIN3 +  means cervical intraepithelial neoplasia grade 3 or worse. PPV means positive predictive value. NPV means negative predictive value.

## Discussion

This was the first large‐scale cross‐sectional study on the application of HPV testing with type 16/18 genotyping for cervical cancer screening in Chinese women. Using pathologic diagnosis of colposcopic biopsy as a standard, we assessed the effectiveness of HPV testing, with cytology as a control, to identify high‐grade CIN. Considering that cytological diagnosis significantly relies on cytologists, we employed at least two cytologists to blindly read the same slide to ensure the accuracy of cytological diagnosis. Our results revealed that Youden's index (YI) of HPV detecting CIN2+ and CIN3+ was higher than that of cytology after bias correction (0.81 vs. 0.64, 0.82 vs. 0.71) with sensitivities of 90.0% and 91.3%, respectively, which were significantly higher than that of cytology (66.7% and 73.9%), and specificities of 91.3% and 90.9%, respectively, which were slightly lower, yet still acceptable, than cytology (97.2% and 97.0%). Our results are consistent with previous studies on HPV testing in areas other than China 5, 14, 15, suggesting that HPV testing demonstrates effective performance in detecting high‐grade CIN and provides more assuring negative results than cytology. Because the results of HPV testing do not depend on cytologists, HPV testing is suitable for cervical cancer screening in China and other developing countries where there is a shortage of cytologists.

The HPV prevalence and its distribution in different ages may affect the aptness of HPV testing in cervical cancer screening. HPV testing is not yet recommended for young women less than 25 years old around the world due to the high HPV prevalence in this age period. In our study, the positivity rate of 14 genotypes of high‐risk HPVs was 9.8% in 11,064 women, which was consistent with 10.4% of the worldwide prevalence 16. After age stratification, the positivity rate in the 21‐ to 24‐year group was as high as 15.4%, which dropped to 8.5% in the 30‐ to 39‐year group, and then gradually climbed to the second peak of 14.4% in the 60‐ to 65‐year group, which equaled that in the 21‐ to 24‐year group and seemed to be higher than that in European and American women of that age in some reports 4, 17. De SanJose 16 reported the worldwide HPV prevalence via a meta‐analysis and found that the second peak of HPV prevalence in women after 44 years old presented with an area difference. For instance, in European and North American women, the HPV prevalence commonly declines after the age of 25 4, 15, 18, 19, while in Asian and Latin American women, the second rise in the HPV prevalence may emerge in some areas, such as China 20, 21, Japan 22, 23, Chile 24, Colombia 25, and Mexico 26. Some factors may be related to the second peak in the HPV prevalence, such as increased extramarital sexual behaviors of their husbands or themselves, or decreased immunity due to a female hormone decline during peri‐ or post‐menopause, which could lead to potential activation of HPV with a low replication status 16, 27, 28. To explore whether HPV testing was suitable for Chinese women aged 50 or above with a high prevalence of HPV, we divided the population into 21‐ to 24‐, 25‐ to 49‐, and 50‐ to 65‐year groups. Since there were too few samples in the 21‐ to 24‐year group because many women were going out for work or study, or were unmarried, we excluded them from the analysis. Our results showed that the sensitivity of HPV testing in the 50‐ to 65‐year group was higher than that in the 25‐ to 49‐year group, but the difference was not significant, while the specificity was significantly lower than that in the 25‐ to 49‐year group. The NPV and PPV of HPV detecting high‐grade CIN also revealed similar differences between the 50–65‐ and 25‐ to 49‐year groups. Our findings suggest that primary HPV testing could have a higher false‐positive proportion for detecting high‐grade CIN in peri‐ or post‐menopausal Chinese women, which probably results in more unnecessary colposcopies. It may be valuable to further study whether HPV‐based screening is suitable in peri‐ or post‐menopausal women who live in the area where the second peak of HPV prevalence emerges.

HPV or cytology testing alone has insufficient sensitivity or specificity, but the combination of two techniques can strengthen the detection advantage and overcome each test's individual weaknesses, elevating the accuracy of screening 29, 30. With HPV and cytology co‐testing screening as a control strategy, we found that the strategy of primary cytology with HPV testing triage, because its standard for referring to colposcopy was the same as co‐testing (cytology LSIL or worse, or HPV+/cytology ASC‐US), showed equal effectiveness and number of colposcopies while consuming only half of the tests to detect one case of high‐grade CIN compared to co‐testing. The strategy of primary HPV testing with cytology triage revealed the highest specificity and PPV, resulting in fewer performed cytology tests and colposcopies to detect one case of high‐risk CIN, but it showed the lowest sensitivity and NPV in the first round of screening, which may be associated with poor effectiveness of triage by cytology due to its low sensitivity. It has been reported that HPV 16/18 genotyping demonstrates optimal triage performance in American women who are HPV positive 5, 7, 31, 32. In the study, we also found that primary HPV testing with type 16/18 genotyping showed the highest sensitivity and NPV and promised similar specificity and PPV for detecting high‐grade CIN compared with co‐testing screening. Importantly, this strategy further reduced the number of necessary cytology tests compared to other HPV testing techniques, requiring only 7.0 or 10.4 tests to detect one case of CIN 2+ or CIN3+, respectively. Therefore, the strategy of primary HPV testing with type 16/18 genotyping may be more feasible than other strategies in China or other developing countries where screening is usually unruled and nonstandard and cytologists are lacking.

Since our study is cross‐sectional, our conclusions are preliminary, and there may be bias. For instance, Strategy 4 sends HPV 16/18 cases with NILM cytology directly to colposcopy, whereas these women in Strategy 3 will be sent to colposcopy one year later, and some of them will not need colposcopy in the second round of screening because of transient HPV16/18 infection. Therefore, the sensitivity of Strategy 3 will be considerably increased, while the PPV of Strategy 4 will be considerably decreased by follow‐up performed on HPV positive/NILM women. It has been observed that direct referral of HPV 16/18 positive women to colposcopy is possibly an excessive treatment.

Considering our results together, HPV testing has optimal effectiveness for detecting high‐grade CIN and is suitable for cervical cancer screening in Chinese women who are older than 25 years of age. The strategy of primary HPV testing with type 16/18 genotyping shows the highest sensitivity and NPV with similar specificity and PPV among different screening strategies, and it requires fewer performed cytology tests and colposcopies to detect one case of high‐grade CIN, suggesting that this strategy possesses optimal cost/effectiveness in the first round of screening and is a feasible strategy of cervical cancer screening for Chinese women.

## Conflict of Interest

None declared.
